# RNAseq analysis of hippocampal microglia after kainic acid-induced seizures

**DOI:** 10.1186/s13041-018-0376-5

**Published:** 2018-06-20

**Authors:** Dale B. Bosco, Jiaying Zheng, Zhiyan Xu, Jiyun Peng, Ukpong B. Eyo, Ke Tang, Cheng Yan, Jun Huang, Lijie Feng, Gongxiong Wu, Jason R. Richardson, Hui Wang, Long-Jun Wu

**Affiliations:** 1 Department of Neurology, Mayo Clinic, 200 First Street SW, Rochester, MN 55905 USA; 2 Department of Pharmacology, School of Pharmacy, Nantong University, 19 Qixiu Road, Nantong, 226001 Jiangsu China; 3 Admera Health LLC, South Plainfield, NJ 07080 USA; 4 Department of Histology and Embryology, School of Basic Medical Sciences, Anhui Medical University, Hefei, 230032 Anhui China; 5 One Harvard Street Institute of Health, Brookline, MA 02446 USA; 6 Department of Pharmaceutical Sciences and Center for Neurodegenerative Disease and Aging, Northeast Ohio Medical University, Rootstown, OH 44272 USA; 7 Department of Neuroscience and Cell Biology, Rutgers-Robert Wood Johnson Medical School, Piscataway, NJ 08854 USA; 8 Department of Neuroscience, Mayo Clinic, Jacksonville, FL 32224 USA

## Abstract

Microglia have been shown to be of critical importance to the progression of temporal lobe epilepsy. However, the broad transcriptional changes that these cells undergo following seizure induction is not well understood. As such, we utilized RNAseq analysis upon microglia isolated from the hippocampus to determine expression pattern alterations following kainic acid induced seizure. We determined that microglia undergo dramatic changes to their expression patterns, particularly with regard to mitochondrial activity and metabolism. We also observed that microglia initiate immunological activity, specifically increasing interferon beta responsiveness. Our results provide novel insights into microglia transcriptional regulation following acute seizures and suggest potential therapeutic targets specifically in microglia for the treatment of seizures and epilepsy.

**Electronic supplementary material:**

The online version of this article (10.1186/s13041-018-0376-5) contains supplementary material, which is available to authorized users.

## Introduction

Temporal lobe epilepsy (TLE) represents the most common form of focal epileptic disorder. While several pharmaceutical treatments are currently available to mitigate and reduce seizure occurrence, as many as one third of patients display resistance to medication [1]. As such, an unmet need exists, requiring further investigation into the mechanisms underlying TLE. The rodent kainic acid (KA) epilepsy model can recapitulate many of the physical features of TLE including behavioral seizures and neuropathological lesions [2]. Therefore, many investigations have focused on how KA alters the activity and viability of neurons. However, comparatively little attention has been paid to glial cells, including astrocytes and microglia, in epileptogenesis [3, 4].

Comprising between 5 and 15% of total central nervous system (CNS) cells, microglia predominantly serve as the resident immune cell of the CNS. Recent evidence has also revealed that microglia have a diverse set of roles within the CNS, including directing neuronal maturation and supporting synaptic turnover [5, 6]. With regard to epilepsy, it was established relatively early that large numbers of reactive microglia can be found within the hippocampus of temporal lobe epilepsy patients [7, 8]. Our recent studies demonstrated that seizures can acutely induce microglia-neuron interaction as well as the changes in microglial landscape [9–12]. Microgliosis and inflammatory cytokine release has been observed within areas of neuronal damage implicating microglia in promotion of neuropathy [13]. However, microglia may also have neuroprotective roles such as modulating excitotoxicity.

Since microglia seem to be an important part of the epileptic response, we investigated how KA-induced seizures modulate microglial transcriptional activity and alters their phenotype. Specifically, we investigated hippocampal microglia since this brain region is one of the most affected by seizure [14]. To explore this, we performed RNAseq analysis, a powerful tool to determine wide scale phenotypic alterations, on isolated hippocampal microglia from mice that received KA. We report that KA-induced seizures resulted in significant transcriptional changes to microglia when compared to sham controls. Specifically, there are significant increases in the expression of metabolic and mitochondrial pathways. Coincidently, we observed that immune related factors were also being up-regulated, including several chemokine factors such as chemokine ligand 5 (CCL5) and C-X-C motif chemokine 10 (CXCL10). We also observed that microglia increased their responsiveness to interferon β, possibly through interferon regulatory factor 7 (Irf7). Thus, we show that KA-induced seizures significantly regulate the microglia transcriptome, providing novel directions for further investigation.

## Results

### Kainic acid induced seizures significantly alters microglial gene expression profile

**Fig. 1:**
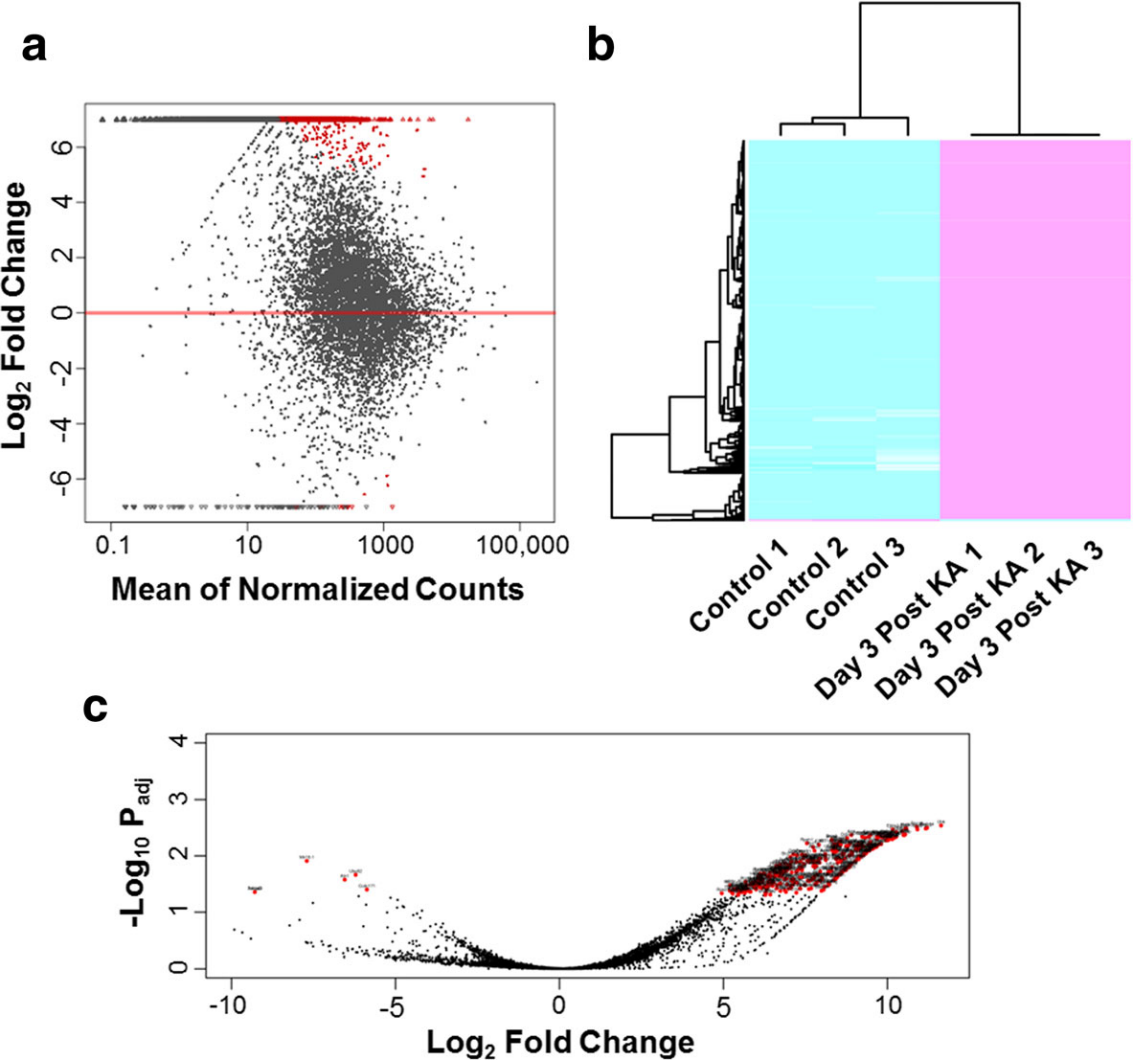
Differentially expressed genes between the sham control and KA treated groups. **a** MA-plot of gene expression. All significant differentially expressed genes (P_adj_ < 0.05) and locally weighted smoothing (LOESS) line are colored in red. **b** Heat map and hierarchical clustering was performed based on all differentially expressed genes. Magenta indicates high relative expression, and cyan indicates low relative expression. **c** Volcano plot of gene expression. All significant differentially expressed genes are colored in red and labeled by gene symbols

To begin our investigation, heterozygote CX3CR1^GFP/+^ mice were treated with kainic acid (KA) via ICV injection to induce an acute seizure response [12]. Microglia in the mouse hippocampus show dramatic reactivity following KA-induced seizure strating at as early as 1 day and peaks at 3 days after KA treatment [15]. We therefore focused on hippocampus microglia isolated via FACS 3 days after KA-induced seizures. RNAseq libraries were constructed using the isolated cells and loaded onto an Illumina Hiseq platform. DEseq was used to determine differential gene expression. From the results, over 2300 differentially expressed genes were identified (Fig. 1a, Additional file 1: Table S1). Of these, we observed many of the suggested microglia specific genes including P2Y12, Tmem119, and Olfml3 [16]. Additionally, we detected only slight increases to myelin (e.g.*,* PLP), neuronal (e.g.*,* Rbfox3, Map2), and astrocytes (e.g.*,* Gfap, Aldh1l1) markers within samples isolated from KA treated mice, with only GFAP registering as significant. These factors were not detected within the control samples. Since it has been suggested that the phagocytic capacity of microglia is substantially reduced following KA-seizure [17] and that microglial could express GFAP [18] we believe that the genes alterations that were deemed significant reflect microglia specific alterations. These results demonstrated the purity of microglia sorting. The overwhelming majority of differentially expressed genes were up-regulated in the microglia samples from KA treated mice with few genes being down-regulated when compared to sham controls (Fig. 1b-c). Table 1 lists the top 25 up-regulated and Table 2 the identified down-regulated genes. Table 3 lists the top 25 genes found only in the KA-treated animals as determined by P_adj_ values.

**Table 1:** Top 25 most up-regulated genes

ENSEMBL	Gene ID	Gene Symbol	Gene Name	Log_2_ Fold Change	P_adj_
ENSMUSG00000019505	22187	Ubb	ubiquitin B(Ubb)	11.64	2.89E-03
ENSMUSG00000006418	81018	Rnf114	ring finger protein 114(Rnf114)	11.21	3.18E-03
ENSMUSG00000005881	66366	Ergic3	ERGIC and golgi 3(Ergic3)	11.17	3.27E-03
ENSMUSG00000090841	17904	Myl6	myosin, light polypeptide 6, alkali, smooth muscle and non- muscle(Myl6)	10.91	3.39E-03
ENSMUSG00000040952	20085	Rps19	ribosomal protein S19(Rps19)	10.90	3.07E-03
ENSMUSG00000042650	268420	Alkbh5	alkB homolog 5, RNA demethylase(Alkbh5)	10.58	3.27E-03
ENSMUSG00000047215	20005	Rpl9	ribosomal protein L9(Rpl9)	10.54	3.27E-03
ENSMUSG00000020664	13382	Dld	dihydrolipoamide dehydrogenase(Dld)	10.54	4.14E-03
ENSMUSG00000025959	93691	Klf7	Kruppel-like factor 7 (ubiquitous)(Klf7)	10.52	4.28E-03
ENSMUSG00000022982	20655	Sod1	superoxide dismutase 1, soluble(Sod1)	10.51	4.21E-03
ENSMUSG00000026213	71728	Stk11ip	serine/threonine kinase 11 interacting protein(Stk11ip)	10.49	4.28E-03
ENSMUSG00000031483	244373	Erlin2	ER lipid raft associated 2(Erlin2)	10.45	3.66E-03
ENSMUSG00000029298	236573	Gbp9	guanylate-binding protein 9(Gbp9)	10.27	4.58E-03
ENSMUSG00000034855	15945	Cxcl 10	chemokine (C-X-C motif) ligand 10(Cxcl10)	10.27	3.78E-03
ENSMUSG00000070031	434484	Sp140	Sp140 nuclear body protein(Sp140)	10.24	4.59E-03
ENSMUSG00000054920	71778	Klhl5	kelch-like 5(Klhl5)	10.22	4.74E-03
ENSMUSG00000040447	216892	Spns2	spinster homolog 2(Spns2)	10.17	4.91E-03
ENSMUSG00000022884	13682	Eif4a2	eukaryotic translation initiation factor 4A2(Eif4a2)	10.16	3.39E-03
ENSMUSG00000028962	20535	Slc4a2	solute carrier family 4 (anion exchanger), member 2(Slc4a2)	10.15	4.93E-03
ENSMUSG00000047153	219094	Khnyn	KH and NYN domain containing(Khnyn)	10.15	5.07E-03
ENSMUSG00000030298	110379	Sec13	SEC13 homolog, nuclear pore and COPII coat complex component(Sec13)	10.12	4.98E-03
ENSMUSG00000031378	11666	Abcd1	ATP-binding cassette, sub-family D (ALD), member 1(Abcd1)	10.11	4.28E-03
ENSMUSG00000004568	102098	Arhgef18	rho/rac guanine nucleotide exchange factor (GEF) 18(Arhgef18)	10.02	5.27E-03
ENSMUSG00000030577	12483	Cd22	CD22 antigen(Cd22)	10.02	5.22E-03
ENSMUSG00000031858	74549	Mau2	MAU2 sister chromatid cohesion factor(Mau2)	10.01	4.58E-03

**Table 2:** Down-regulated genes

ENSEMBL	Gene ID	Gene Symbol	Gene Name	Log_2_ Fold Change	P_adj_
ENSMUSG00000000562	11542	Ccdc171	adenosine A3 receptor(Adora3)	−5.87	3.98E-02
ENSMUSG00000090137	22186	Uba52	ubiquitin A-52 residue ribosomal protein fusion product 1(Uba52)	−6.22	2.17E-02
ENSMUSG00000052407	320226	Atn1	coiled-coil domain containing 171(Ccdc171)	−6.55	2.65E-02
ENSMUSG00000092995	387134	Mir16–1	microRNA 16–1(Mir16–1)	−7.71	1.23E-02
ENSMUSG00000004263	13498	Adora3	atrophin 1(Atn1)	−9.29	4.39E-02
ENSMUSG00000074344	69296	Tmigd3	transmembrane and immunoglobulin domain containing 3(Tmigd3)	−9.29	4.39E-02

**Table 3:** Top 25 differentially expressed genes only observed in KA treated group

ENSEMBL	Gene ID	Gene Symbol	Gene Name	P_adj_
ENSMUSG00000069516	17105	Lyz2	lysozyme 2	6.62E-04
ENSMUSG00000060938	19941	Rpl26	ribosomal protein L26	1.15E-03
ENSMUSG00000002602	26362	Axl	AXL receptor tyrosine kinase	2.25E-03
ENSMUSG00000031320	20102	Rps4x	ribosomal protein S4, X-linked	2.25E-03
ENSMUSG00000049313	20660	Sorl 1	sortilin-related receptor, LDLR class A repeats-containing	2.25E-03
ENSMUSG00000062006	68436	Rpl34	ribosomal protein L34	2.25E-03
ENSMUSG00000063524	619547	Rpl34-ps1	ribosomal protein L34, pseudogene 1	2.25E-03
ENSMUSG00000069516	100043876	Gm4705	predicted gene 4705	2.25E-03
ENSMUSG00000063524	13806	Eno1	enolase 1, alpha non-neuron	2.25E-03
ENSMUSG00000069892	245240	9,930,111 J21 Rik2	RIKEN cDNA 9,930,111 J21 gene 2	2.25E-03
ENSMUSG00000089809	319818	A930011G23Rik	RIKEN cDNA A930011G23 gene	2.25E-03
ENSMUSG00000090733	57294	Rps27	ribosomal protein S27	2.25E-03
ENSMUSG00000073418	12268	C4b	complement component 4B	2.83E-03
ENSMUSG00000001794	12336	Capns1	calpain, small subunit 1	2.94E-03
ENSMUSG00000003518	72349	Dusp3	dual specificity phosphatase 3	2.94E-03
ENSMUSG00000005566	21849	Trim28	tripartite motif-containing 28	2.94E-03
ENSMUSG00000009687	18301	Fxyd5	FXYD domain-containing ion transport regulator 5	2.94E-03
ENSMUSG00000022415	20972	Syngr1	synaptogyrin 1	2.94E-03
ENSMUSG00000022477	11429	Aco2	aconitase 2, mitochondrial	2.94E-03
ENSMUSG00000022565	18810	Plec	plectin	2.94E-03
ENSMUSG00000024679	68774	Ms4a6d	membrane-spanning 4-domains, subfamily A, member 6D	2.94E-03
ENSMUSG00000025498	54123	Irf7	interferon regulatory factor 7	2.94E-03
ENSMUSG00000026222	20684	Sp100	nuclear antigen Sp100	2.94E-03
ENSMUSG00000026430	54354	Rassf5	Ras association (RalGDS/AF-6) domain family member 5	2.94E-03
ENSMUSG00000034854	73822	Mfsd12	major facilitator superfamily domain containing 12	2.94E-03

**Fig. 2:**
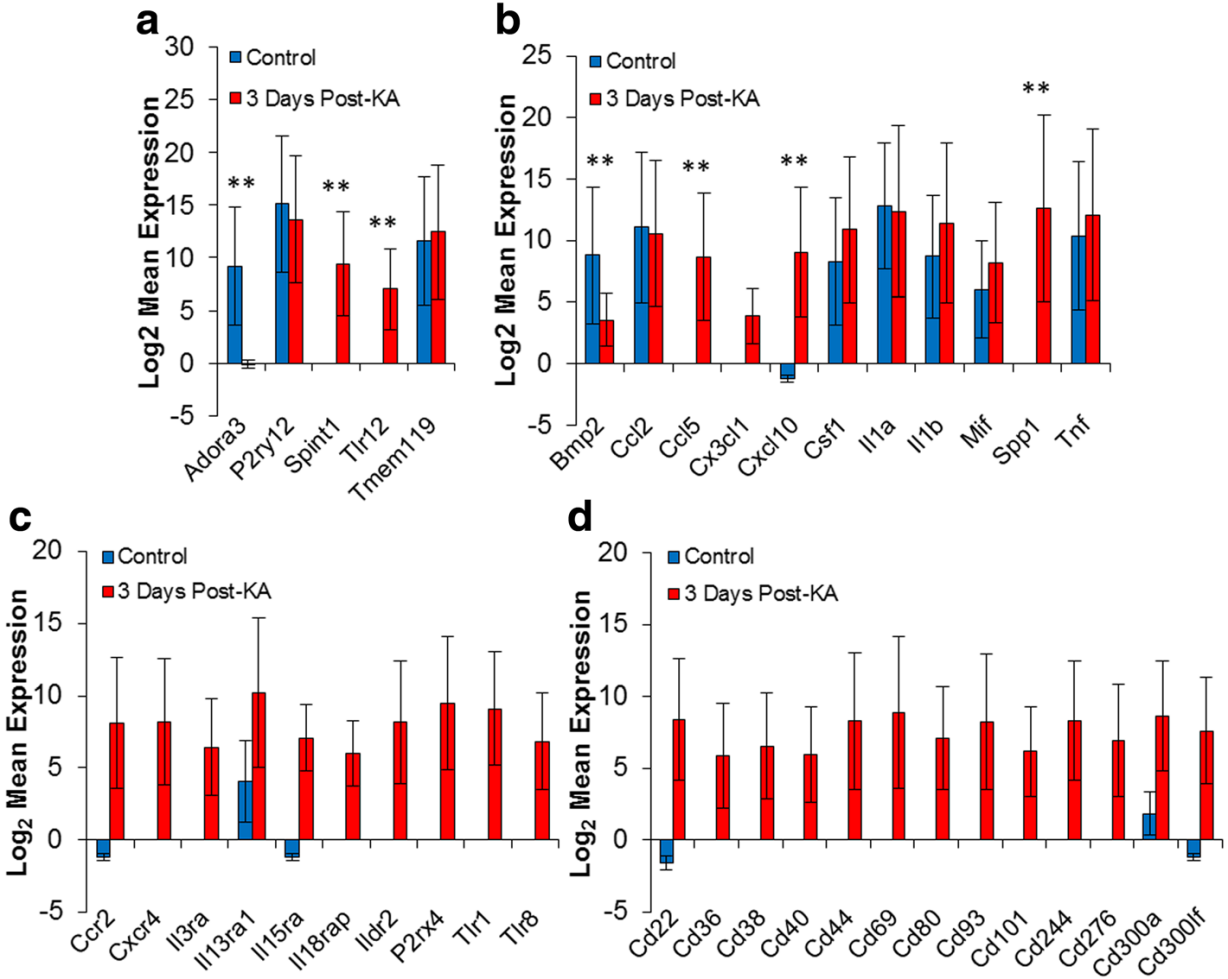
Selected differential expressed genes. Expression results were investigated for genes relating to microglial specificity and inflammatory and immunological regulation. **a** Microglial markers. **b** Secreted factors. **c** Related receptors. **d** CD markers. Values are expressed and mean ± standard error. **P_adj_ < 0.05. All gene listed in panel (**c** and **d**) had a P_adj_ < 0.05

We next determined whether KA-induced seizures affected microglial specific markers. Using the list determined by Hickman et al. [16]*,* we found that seven of the listed microglial markers were differentially expressed (Fig. 2a, Additional file 2: Figure S1). These were adenosine A3 receptor (Adora 3), crystallin beta A4 (Cryba4), galactose-3-O-sulfotransferase 4 (Gal3st4), lipase member H (Liph), membrane-spanning 4-domains, subfamily A, member 6B (Ms4a6b), serine peptidase inhibitor Kunitz type 1 (Spint1), and toll-like receptor 12 (Tlr12). Since KA treatment has also been shown to induce inflammatory responses [15], we also investigated our list of differentially expressed genes for potential inflammatory markers. Indeed, we found a number of inflammatory factors are increased within microglia isolated from KA treated mice, including C-C motif chemokine ligand 5 (Ccl5), Ccl7, and C-X-C motif chemokine ligand 10 (Cxcl10) (Fig. 2b, Additional file 2: Figure S2). We determined that expression of several inflammatory and immunological response receptors are also increased (Fig. 2c). These receptors included C-C motif chemokine receptor 2 (Ccr2), C-X-C motif chemokine receptor 4 (Cxcr4), and Tlr1. Finally, a significant number cluster of differentiation (CD) markers were significantly increased (Fig. 2d). The majority of identified CD markers are related to immunological responses including CD40, CD69, and CD80 [19, 20]. These results suggest that microglia are undergoing immunological activation in response to KA-induced seizures.

### Gene ontology analysis indicates significant increases to metabolic processes

**Fig. 3:**
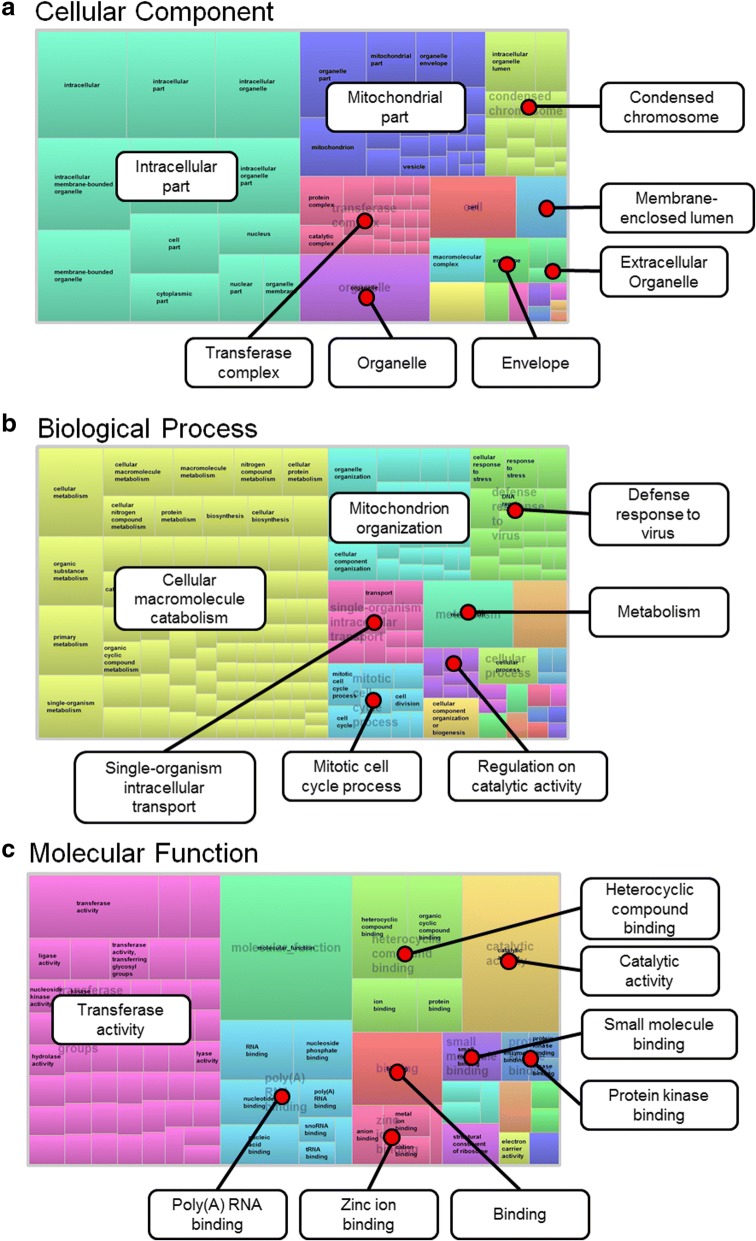
Functional classification of the differentially expressed genes. **a** Cellular component. **b** Biological process. **c** Molecular function. Visualization of identified Gene Ontology terms was completed using REViGO [22]. Analysis was run using the P_adj_ for each identified term, medium allowed similarity (0.7), and SimRel similarity measurement. Individual term size weight within each TreeMap was determined by associated P_adj_.

Our next step was to identify if any unifying features existed within our differential expression data set. As such, we utilized clusterProfiler to perform gene ontology (GO) analysis [21]. We investigated our data set using the three major classifications, cellular component, biological process, and molecular function (Additional file 3: Table S2, Additional file 4: Table S3 and Additional file 5: Table S4). To further visualize our results, identified GO terms were input into REViGO [22]. This web-based application allows for long lists of GO terms to be summarized and grouped based on semantic similarities. REViGO analysis was run using the associated P_adj_ for each identified GO term, with medium allowed similarity (0.7), and SimRel similarity measurement. TreeMaps were then generated for each ontology classification. Each box represents GO terms that are then grouped and colored based on keyword similarities. Box size indicates each terms level of significance as determined by input P_adj_ values. Added labels highlight overarching grouping terms. As Fig. 3a illustrates there are significant alterations to intracellular factor expression, especially within the mitochondria. Moreover, Biological process GO analysis showed that there seems to be significant alterations to microglial metabolism, with catabolism being at the forefront (Fig. 3b). It also identified that microglia were activating viral defense mechanisms following seizure. Finally, we observed that a number of transferase activities were being undertaken following seizure (Fig. 3c).

### Kainic acid treatment may sensitize microglia to interferon beta

Delving deeper into the identified GO terms it was observed that a number of related terms were pertinent to type I interferons, specifically interferon β (IFN-β). Table 4 summarizes these identified GO terms. IFN-β is a type-I interferon that binds interferon-α/β receptor (IFNAR) to regulate a multitude of signaling cascades particularly the JAK/STAT pathway [23]. IFN-β has also been suggested to modulate microglial activity in multiple sclerosis and pathological neovascularization [24, 25]. Since IFN-β signaling was well represented within our GO analysis, we believe that IFN-β is important to the microglial modulation that occurs following KA-induced seizures.

**Table 4:** Type I interferon related GO terms

GO Term ID	Term Name	P_adj_
GO:0032480	negative regulation of type I interferon production	0.0012
GO:0032479	regulation of type I interferon production	0.0019
GO:0034340	response to type I interferon	0.0046
GO:0032606	type I interferon production	0.0055
GO:0032648	regulation of interferon-beta production	0.0109
GO:0032608	interferon-beta production	0.0166
GO:0035456	response to interferon-beta	0.0173
GO:0060337	type I interferon signaling pathway	0.0204
GO:0071357	cellular response to type I interferon	0.0204
GO:0032688	negative regulation of interferon-beta production	0.0375
GO:0060340	positive regulation of type I interferon-mediated signaling pathway	0.0375
GO:0035458	cellular response to interferon-beta	0.0429
GO:0060338	regulation of type I interferon-mediated signaling pathway	0.0497

### Pathway analysis reveals both metabolic and immune response processes are altered

**Fig. 4:**
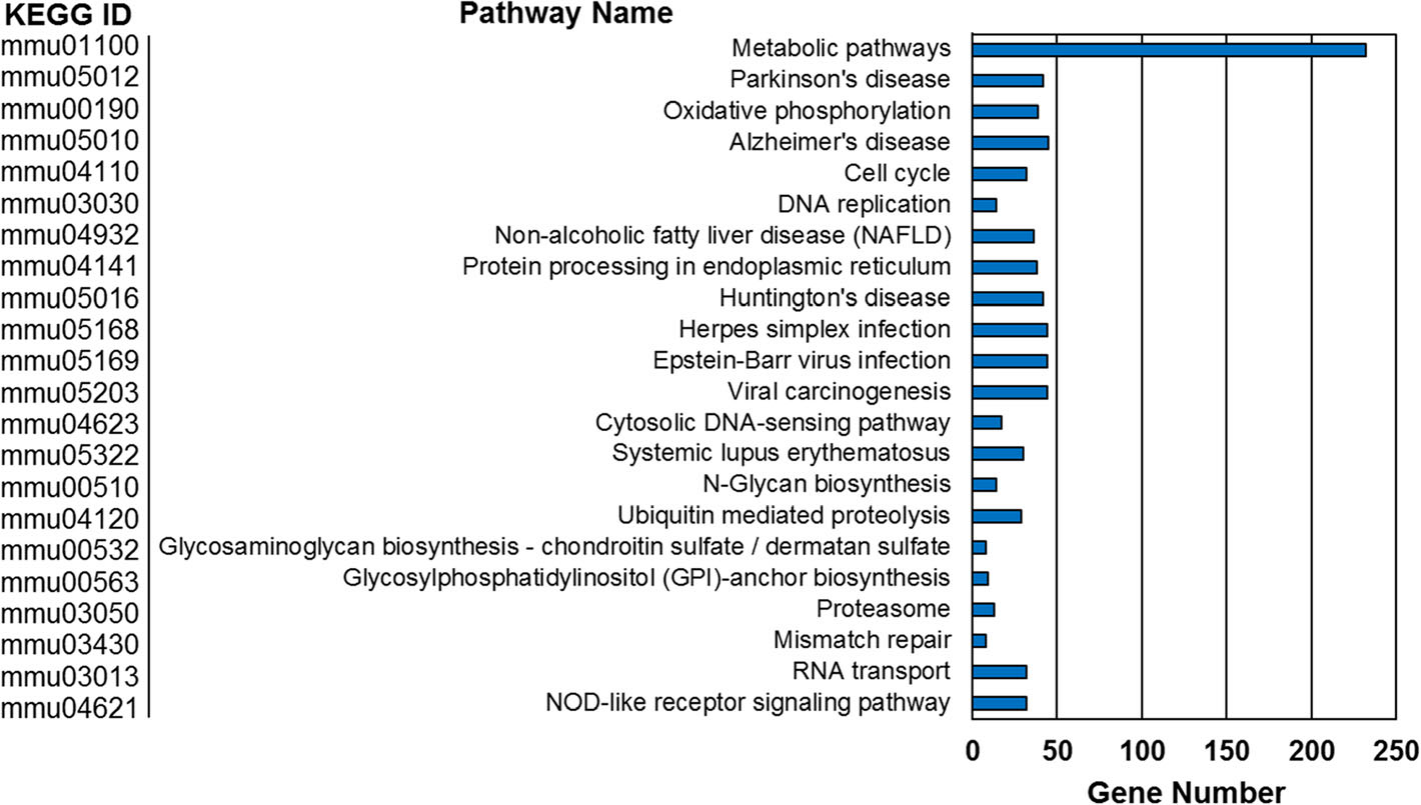
Pathway enrichment of differentially expressed genes. KEGG pathway enrichment of up-regulated genes following KA treatment with a q-value < 0.05

**Fig. 5:**
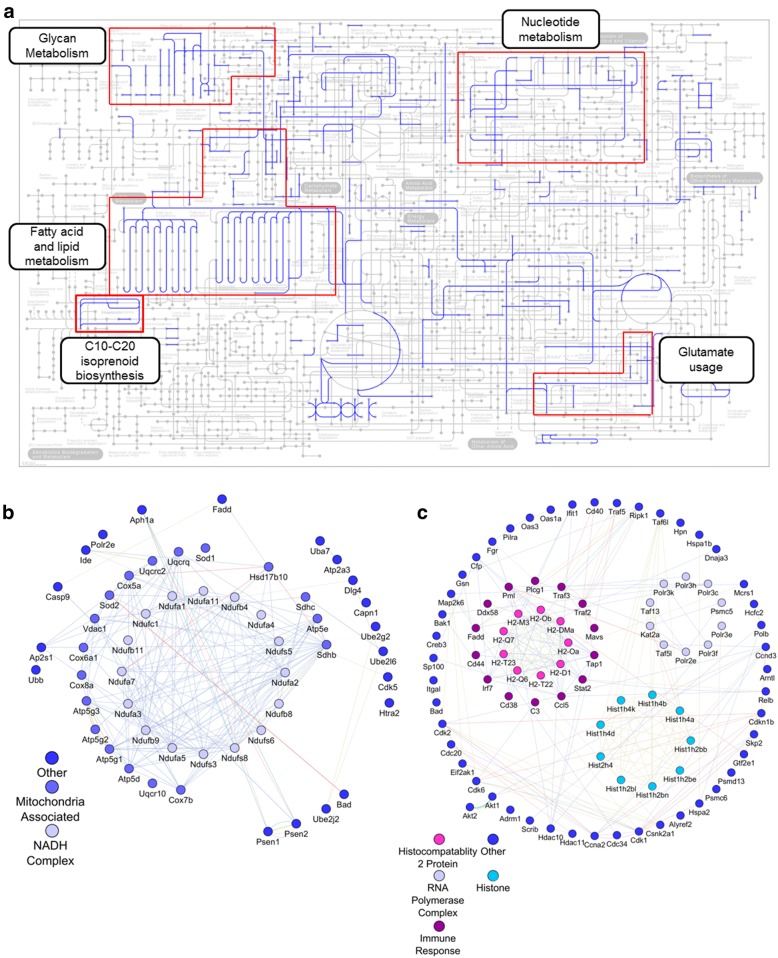
Functional analysis of genes from identified pathways. **a** KEGG mapper was utilized to determine associated metabolic pathways. Differentially expressed genes relating to the identified (**b**) neurodegenerative and (**c**) viral pathways were loaded into GeneMANIA [26] to generate putative interaction diagrams. Displayed interactions were limited to only experimentally determined relations

Finally, we performed pathway analysis on the differential expression data set using the clusterProfiler enrichKEGG function (Fig. 4). Unsurprisingly, this analysis corroborated our GO analysis results in that metabolism was significantly enriched in our data set. We also identified several pathways relating to neurological diseases (i.e.*,* Parkinson’s, Alzheimer’s, and Huntington’s disease) (Fig. 4). Using KEGGmapper we were able to further investigate which specific metabolic pathway were being affected. We found that Glycan, fatty acid and lipid, and nucleotide metabolism are all up-regulated within the KA treated samples. Moreover, we observed several pathways involving glutamate utilization and isoprenoid biosynthesis were also affected (Fig. 5a).

While metabolism was by far the most significantly altered pathway term identified, several other pathways of note were identified, specifically those relating to neurodegenerative diseases (i.e.*,* Parkinson’s, Huntington’s, Alzheimer’s) and viral response (i.e.*,* Herpes simplex, Epstein-Barr, viral carcinogenesis). While it was consistent with our exploration avenue to observe pathways relating to neurodegenerative diseases, we observed viral responses in both GO and pathway analysis. As such, we further explored the gene relationships underlying these identified pathway terms. Differential expressed genes identified to be part of the indicated KEGG pathway terms were analyzed with the GeneMANIA application for Cytoscape V3.5.1 [26]. GeneMANIA utilizes both published information and computational predictions to identify relationships between input genes. It will also suggest possible interaction partners not initially input into the query. Indeed, we demonstrate that the overwhelming majority of genes associated with the identified neurodegenerative pathways were related to mitochondrial function, specifically the electron transport chain (Fig. 5b). This is consistent with our GO analysis. Investigation of viral pathway term genes however revealed a more diverse set of groupings (Fig. 5c). These include genes related to RNA polymerase complexes and histones. Both of which are consistent with the high levels of transcriptional modulation observed. Additionally, several genes were associated with immunological regulation, such as complement C3, signal transducer and activator of transcription 2 (Stat2), and antigen peptide transporter 1 (Tap1).

## Discussion

The majority of research into epilepsy has focused on neuronal hyperactivities and cell death. However, the role of glia, particularly microglia, in the pathogenesis of epilepsy is an important emerging area of study. Specifically, the transcriptomic alterations of microglia following KA-induced seizure have not been well studied. In this regard, we utilized RNAseq analysis on isolated hippocampal microglia to investigate microglial response during the acute phase after seizure. In total, our results clearly demonstrate that microglia undergo significant alterations following KA-induced seizures, including up-regulation of several inflammatory factors and modulation of mitochondrial activity.

### Microglia may undergo oxidative stress response following KA-induced seizure

The most obvious phenotypic alteration was mitochondrial activity in microglia after seizures. While it is possible that up-regulation of mitochondrial genes is merely indicative of microglia transitioning from a resting to active state, it is also possible that microglia are increasing production of mitochondria-derived reactive oxygen species (ROS). While NADPH oxidase has often been described as the primary source of ROS, it has been well established that NADH dehydrogenase (electron transport chain complex I) can also contribute to ROS formation [27]. Indeed, several complex I subunits (e.g.*,* Ndufs8, Ndufa5, Ndufb8) were differentially expressed in our dataset but no NADPH oxidase subunits were up-regulated. This idea is also supported by the observed up-regulation of superoxide dismutase (Sod) 1 and 2, both of which can convert the superoxide generated by the electron transport chain into hydrogen peroxide [28]. Sod 1 and 2 are critically important for the mitigation of oxidative stress and are altered during epilepsy.

When considering our other results, specifically the observed utilization of glutamate, there is further indication that microglia are responding to oxidative stressors. Our results identified two possible means by which glutamate could be utilized, conversion to either 1) proline or 2) glutathione. Of these, the generation of glutathione may be of significance. From our results we observed differential expression of glutathione peroxidase 3 (Gpx3), glutathione S-transferase omega 1 (Gsto1), glutathione transferase zeta 1 (Gstz1), and glutathione reductase (Gsr) expression, all of which are important to the mitigation of oxidative stress [29, 30]. Understanding the consequence of this response could open new avenues into attenuating oxidative damage following seizures. Moreover, it is interesting that of the four main glutathione peroxidase variants, we only observed increases in Gpx3, which is found within the extracellular space [31]. It is possible that microglia are attempting to mitigate not only their own endogenous oxidative stress but also that within the environment.

### Microglia increase metabolic activity in response to KA-induced seizure

Our results also showed that many genes relating to metabolic activity are significantly up-regulated. Specifically, GO and pathway analysis determined that microglia up-regulated lipid, nucleotide, and glycan metabolism. These metabolic activities have also been observed during transcriptional analysis of total rat cortex following sarin-induced seizure [32]. We also identified that geranylgeranyl diphosphate synthase 1 (Ggps1) was up-regulated. Ggps1 is responsible for synthesis of the isoprenoid intermediate geranylgeranyl diphosphate (GGPP), which can be attached to a wide assortment of proteins via geranylgeranyltransferases (GGT) like Rab GGT, whose alpha subunit was differentially expressed in our data set [33, 34]. Within Alzheimer’s disease it was shown that GGPP may influence microglial inflammatory response via modulation of Rho GTPase [35]. Moreover, many of the positive effects of statin drugs (e.g.*,* reducing excitotoxicity and inflammation) within Alzheimer’s disease, Parkinson’s disease, and multiple sclerosis could be attributed to mitigation of isoprenoid intermediates, like GGPP [36–39]. The observed mitigation of KA induced seizure symptoms by statins may also involve similar mechanisms [40]. Given our results, it would be of interest to determine if statins improve seizure recovery by attenuating microglial inflammatory response.

### Microglia undergo immunological activation in response to KA-induced seizure

Microglia as a principal immune cell in the brain are activated in human epileptic brain and rodent seizure models [4]. Not surprisingly, we also observed that microglia underwent immunological activation, as seen by enrichment of viral response pathways, at 3 days post KA-induced seizures in mice. However, underlying each of these pathways was a shared set of up-regulated genes, including a number of histocompatibility genes, many of which seem to correlate with non-classical major histocompatibility complexes. More specifically, we observed that H2-T23, which encodes Qa-1, and several genes that make up Qa-2 (i.e.*,* H2-Q6, H2-Q7, and H2-Q8) were differentially expressed within our data set [41, 42]. These histocompatibility complexes have been shown to modulate the activity of natural killer (NK) cells [43, 44]. In regards to neuroinflammation, it was reported that the soluble forms of MHC-E and MHC-G might be related to inflammation protection within multiple sclerosis [45]. However, very little has been done to investigate the roles of these histocompatibility complexes within epilepsy. Given the indications that NK cells are increased following temporal lobe epilepsy, it is worth investigating whether microglia are modulating NK cell activity within the hippocampal region following seizure induction, and whether this modulation is inhibitory of stimulatory.

### Interferon beta may modulate microglial activity following KA-induced seizure

Another sign of microglial immunomodulation was the identification of a number of IFN-β responsive terms during GO analysis. IFN-β is typically seen as being anti-inflammatory and has become a common treatment option for relapsing-remitting multiple sclerosis patients [46]. However, there are also indications that type-I interferons may negatively regulate brain activity during aging [47]. Thus, IFN-β may have differential roles depending on disease context. In relation to microglia it has been shown that IFN-β can induce chemokine CCL5 expression, which was highly up-regulated in our data set and in our recent cytokine array [15] after KA-induced seizures. It has also been shown that interferon regulatory factor 7 (Irf7), which is suggested to be the master regulator of type-I interferon-dependent immune response, can modulate CCL5 expression [48]. We observed that Irf7 was differently expressed following KA treatment, indicating a possible means by which IFN-β could modulate microglia inflammatory responses following seizures. As a final note, it has been shown that some Irf7 activity may be tightly regulated by non-degenerative ubiquitination [49, 50]. One of the most up-regulated genes in our data set following seizure was ubiquitin (Ubb). Consequently, our data set indicates that several facets of gene regulation are at play within microglia following KA treatment.

### Identified differentially expressed genes warranting further investigation

Finally, while over 2300 differentially expressed genes were identified, we believe that the following selection may be of interest for further investigation. First is osteopontin (secreted phosphoprotein 1; Spp1), which has been observed within neuronal injuries, particularly ischemic stroke [51, 52]. However, little is known about how Spp1 is involved in epileptic seizures even though other less targeted profiling analyses have also noted its up-regulation following seizures [53, 54]. What is known is that its expression seems to be localized to certain areas of the brain, including the CA1 and CA3 regions of the hippocampus [55, 56]. Moreover, it has been suggested that only a sub-set of microglia actively express Spp1, with a possible role in phagocytosis [55]. However, the exact role of Spp1 following epilepsy requires further evaluation.

Next is the adenosine A_3_ receptor (Adora3/A3ar). This gene is of interest as it was the only receptor to be down-regulated within our data set. Adenosine has long been viewed as an endogenous anticonvulsive and will increase dramatically during epileptic seizures [57]. As for Adora3, it was reported that its specific agonist, IB-MECA, could protect against seizures [58]. It was found that Adora3 is highly expressed in microglia and that LPS treatment down-regulates its expression [59]. Moreover, externally induced activation of Adora3 could reduce LPS-induced tumor necrosis factor alpha (TNFα) in both RAW 264.7 macrophages and BV2 microglia [60, 61]. Yet, little else is known about how Adora3 can modulate microglial activity, let alone why we observed a significant down-regulation in expression following KA-induced seizure.

Lastly, while several purinergic receptors have been shown to modulate microglial function during epilepsy, including P2ry12 and P2rx7, we only observed significantly increased expression of P2rx4 [12]. This receptor has been observed to be important to the pathogenesis of several neurological conditions including neuropathic pain and epilepsy [62, 63]. In regards to microglia, P2rx4 expression can be up-regulated via fibronectin, which was differentially expressed in our data set [64]. Within models of neuropathic pain, it has been suggested that activation of P2rx4 induced microglia to release brain-derived neurotropic factors (BDNF), which then affected neuronal activity by modulating GABAergic activity [65, 66]. Since the hippocampus has a significant population of GABAergic interneurons, particularly in the CA1 and CA3 regions, it may be of interest to determine to what extent this crosstalk exists and whether or not blockage of this communication could alleviate seizure symptoms [67].

In conclusion, our results demonstrate that KA-induced seizure acutely affects the phenotypic character of microglia within the hippocampus. Specifically, microglia seem to be undergoing a variety of activations, which could potentially regulate neuronal hyperactivities and seizure behaviors. We have identified a number of mechanisms and gene targets that could provide future directions for therapeutic intervention.

## Methods

### Mice

The described In vivo procedures were approved by Institutional Animal Care and Use Committee (IACUC) in both Rutgers University and Mayo Clinic. We followed the guidelines set forth by the Guide of the Care and Use of Laboratory Animals 8th Edition. Both male and female adult heterozygous microglia GFP reporter mice at two months of age were used. The mice express GFP under control of the fractalkine receptor promoter(CX3CR1^GFP/+^) that selectively label microglia in the CNS [68].

### KA administration

An injection of kainic acid (KA) (Tocris Biosciences, Bristol, UK) via direct intracerebroventricular (ICV) injection to induce seizure was performed as previously described [12, 15]. Briefly, a guide tube (24 gauge) was implanted into CX3CR1^+/GFP^ mice prior to KA injection. After a 24 h recovery period, a 30 gauge needle was inserted through the cannula to deliver the KA solution (0.2 μg in 5 μl). Mice were then observed for induction of seizure response using the method described previously [12]. Briefly, seizure behavior was monitored under a modified Racine scale as follows [12, 15, 69]: (1) freezing behavior; (2) rigid posture with raised tail; (3) continuous head bobbing and forepaws shaking; (4) rearing, falling, and jumping; (5) continuous level 4; and (6) loss of posture and generalized convulsion activity. Mice progressed at least to stage 3 and were sacrificed 3d after seizure. Sham controls did not receive KA administration.

### Microglia isolation

All mice were perfused with ice cold PBS (pH 7.4) 3 days post KA treatment. Hippocampi were excised, minced on ice, and suspended in a trypsin/EDTA solution for 20 mins, in a 37 °C shaker. After incubation, 3 ml DMEM and 50ul DNase was added to the cell suspension. Cell pellets where then suspended in 5 ml HEPES (4-(2- hydroxyethyl)-1- piperazineethanesulfonic acid) buffer, then centrifuged again. Pellets were finally re-suspended in 800 ml HEPES buffer and transferred into a sorting tube on ice. GFP-labeled microglia were isolated via FACS on a MoFlo XDP Cell Sorter (Beckman Coulter, CA, USA). Microglia from sham controls were isolated in the same manner after a corresponding length of time.

### RNAseq analysis

RNA was isolated with the RNeasy Plus Micro Kit (Qiagen, Hilden, Germany). RNA quality was evaluated by Tapestation RNA HS Assay (Agilent Technologies, CA, USA) and Bioanalyzer 2100 Eukaryote Total RNA Nano Kit (Agilent Technologies). Libraries were constructed with the SMART-Seq v4 Ultra Low Input RNA Kit (Takara-Clontech, CA, USA) using manufacturer’s instructions. Final library quantity was determined by KAPA SYBR® FAST qPCR and library quality evaluated by Tapestation RNA HS Assay (Agilent Technologies, CA, USA). Equimolar pooling of libraries were performed based on qPCR values and loaded onto an Illumina Hiseq platform (Illumina, CA, USA).

### Differential gene expression analysis

RNA-seq data were aligned to the mouse reference genome using STAR mapping tool [70]. Read counts were then quantified using HTSeq-count [71]. DESeq, an R Bioconductor package, was used for differential gene expression analysis [72]. It estimates variance-mean dependence in RNA-seq count data and tests for differential expression using a negative binomial distribution model. Heat map and hierarchical clustering of differentially expressed genes was performed using the heatmap function in stats package in R.

### Functional classification of differentially expressed genes

Gene Ontology (GO) analysis is a commonly used approach for functional studies of RNA-seq data. To functional classify the differentially expressed genes between the control and KA treated groups, GO enrichment analysis using clusterProfiler was performed. Additionally, significant KEGG pathways were identified using the enrichKEGG function in clusterProfiler package with FDR < 0.05.

### Statistics

Both KA-treated and control were collected with *n* = 3 mice. The R package DESeq was used on our RNAseq counts to estimate the variance-mean dependence and to test for differential expression. Differentially expressed proteins with adjusted *p*-values < 0.05 using the Benjamini-Hochberg procedure. These proteins were then subjected to pathway enrichment/gene ontology analysis. A Benjamini-Hochberg adjusted *p*-value of < 0.05 was used to identify significantly enriched pathways.

## Additional files

Additional file 1: **Table S1.** List of all identified differentially expressed genes. (XLSX 336 kb)

Additional file 2: **Figure S1.** Expression profiles of microglia specific markers. The expression of microglia specific markers, as determined by Hickman et al. [16] was investigated. The Log_2_ base mean expression of each condition is presented for each gene. Presented error bars are standard error using the Log_2_ standard deviation of each mean. **P_adj_ < 0.05. **Figure S2.** Expression profiles of cytokine markers. The expression of a variety of cytokines was investigated. The Log_2_ base mean expression of each condition is presented for each gene. Presented error bars are standard error using the Log_2_standard deviation of each mean. **P_adj_ < 0.05. (PDF 114 kb)

Additional file 3: **Table S2.** List of all identified biological process GO terms. (XLSX 44 kb)

Additional file 4: **Table S3.** List of all identifed molecular function GO terms. (XLSX 23 kb)

Additional file 5: **Table S4.** List of all identifed cellular compartment GO terms. (XLSX 24 kb)
