# Supplementary figures and images for: RNAseq analysis of hippocampal microglia after kainic acid-induced seizures
Source: Mol Brain. 2018 Jun 20;11:34. doi: 10.1186/s13041-018-0376-5 (PMC6011524; doi:10.1186/s13041-018-0376-5)

FIGURE S1

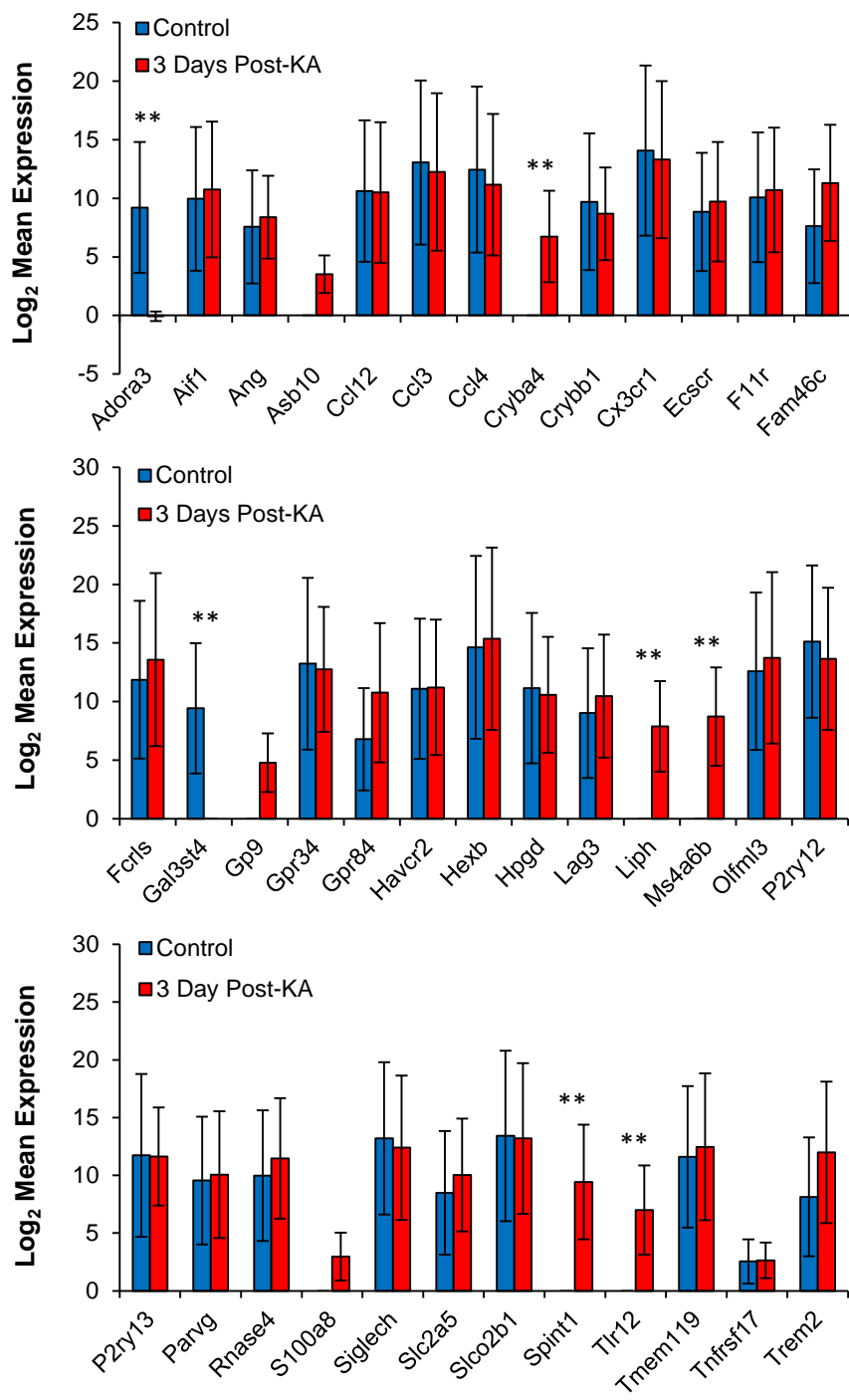

FIGURE S2

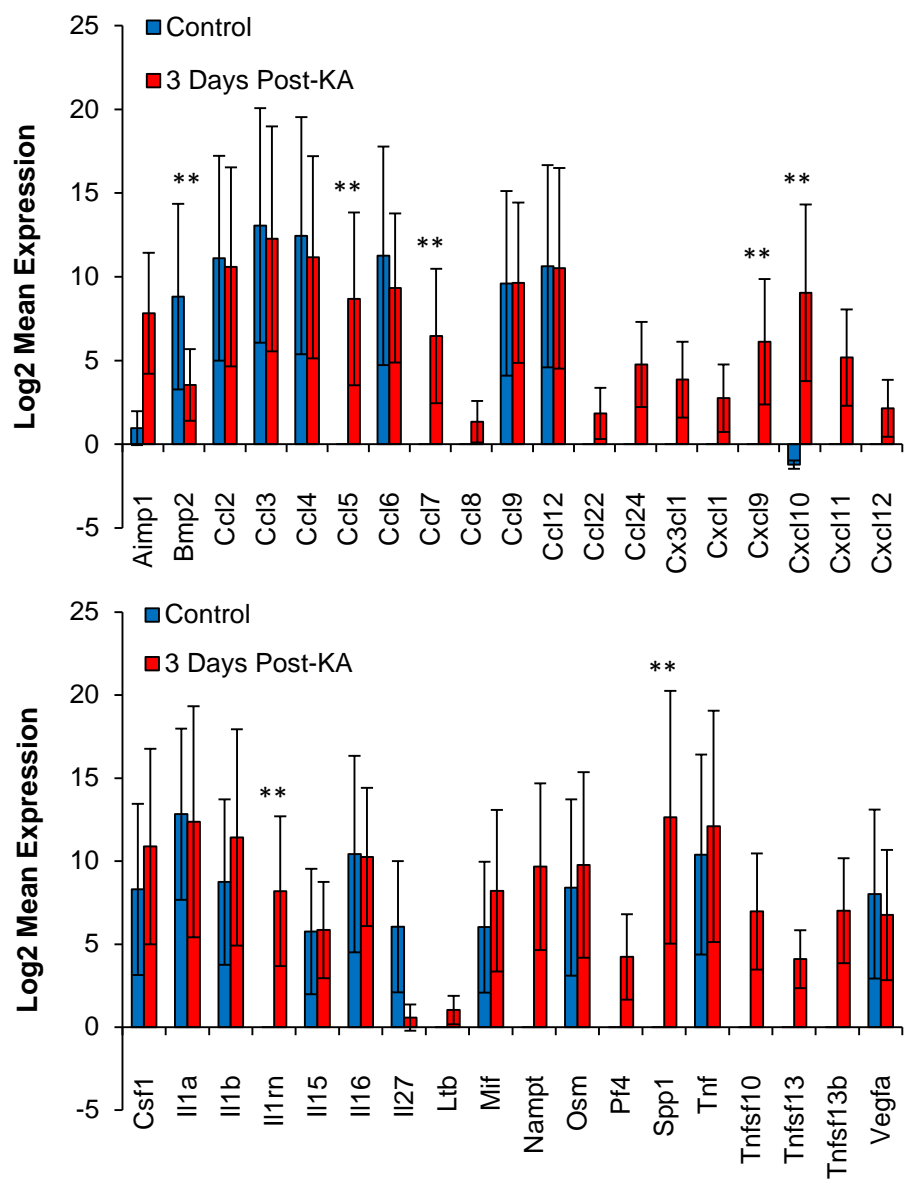

Supplement: Supplementary file 2 — Figure S1. Expression profiles of microglia specific markers. The expression of microglia specific markers, as determined by Hickman et al. [16] was investigated. The Log2 base mean expression of each condition is presented for each gene. Presented error bars are standard error using the Log2 standard deviation of each mean. **Padj < 0.05. Figure S2. Expression profiles of cytokine markers. The expression of a variety of cytokines was investigated. The Log2 base mean expression of each condition is presented for each gene. Presented error bars are standard error using the Log2standard deviation of each mean. **Padj < 0.05. (PDF 114 kb) [file 13041_2018_376_MOESM2_ESM.pdf]
